
JOURNAL INFORMATION
==============================
NLM Title Abbreviation: Med Chir Rev
NLM Journal ID: 0034617

The Medico-Chirurgical Review

Publisher: Biomedical Journal Digitization Project

ARTICLE INFORMATION
==============================
PMCID: PMC4966193
PMID: 29917615
Article version: 1
Subjects: Reviews

Dr. Cuming on the Peripneumony of Children

Print publication date: 1828 Jul 1
Volume: 9
Issue: 17
First page: 67
Last page: 72
1828 May 10
License: This work is free of known copyright restrictions. For more information, please see PMC Back Issue Digitization.
License URL: https://creativecommons.org/publicdomain/mark/1.0/

==============================

be regulated by the mildest means—the diet should be nutritious and plain—and light bitter tonics should be exhibited to improve the digestive process. These means would do more than all the farrago of drugs in Apothecaries' Hall.

We are so convinced of the fact, that a great number of disorders of the general health result from bad air, that we have taken great pains to concentrate Dr. Macculloch's views, and diffuse them widely through the profession. They are calculated to do much good, though some of them may have been carried too far. In succeeding articles we will take up the other subjects treated of in these volumes, and hope to render them still more interesting than the present—which, however, forms an indispensible preliminary to the investigation of malarious diseases generally.

---

### VII.

*Observations on the Peripneumonia of Children.* By THOMAS CUMING, M.D. Assistant Physician to the Institution for Diseases of Children, &c.

[Dublin Transactions, vol. V.]

THERE are few, if any, diseases to which children are more liable, or which prove more fatal, if not timely remedied, than peripneumony. The situation which Dr. Cuming holds enables him to come before the public with ample materials resulting from personal observation, and of these materials we propose to exhibit the more important portions in this article.

In the dissections given by Cheyne and Hastings, the morbid appearances were chiefly confined to the lining membrane of the bronchia; whereas in almost every fatal case, our author found the parenchymatous and mucous tissues equally the seat of inflammatory action. Although the disease occurs at all ages, from a few days to eight or nine years, the most frequent subjects of attack, however, are children between nine months and two years—probably owing to the period of dentition. This disease occurring in children above two years of age, has generally been traced as a sequela of measles or hooping-cough. In some instances, the disease comes on very suddenly. The child goes to bed well, and rises next morning with all the symptoms of well-marked pneumonia. This, however, is rare.

"In general a trifling cough, with other slight symptoms of catarrh, precedes by a day or two the complete formation of the disease. When fully formed, the symptoms by which the disease is characterized are a hurried, laborious, and wheezing respiration; a frequent, short, and dry cough, and a greater or less degree of fever. With the fever are combined an extreme degree of restlessness and impatience, moaning, starting out of sleep, and aversion to be moved. The countenance, though occasionally flushed, is for the most part extremely pallid, sometimes sallow; and as the disease advances it frequently assumes a mottled livid hue, and becomes in some cases swollen and œdematous. When the progress of the malady cannot be stopped, the breathing becomes more hurried and laboured, and the wheezing amounts in many cases to rattling. A state of drowsiness and prostration succeeds to the state of restlessness and pervigilium, which had existed in the beginning; the cough is suspended; the pulse becomes imperceptible; the extremities cold; and in the course of eight or ten days from the commencement of the attack, death generally takes place by suffocation."

The average frequency of natural respiration in children under twelve months, is about 30 in the minute. Dr. C. has often observed it up to 60, 80, 90, or even 100 in the same time. When the respiration comes down from the above to 50 or 40, it indicates recovery. There is sometimes an intermission or irregularity in the breathing. That the breathing is laborious may readily be inferred, from the heaving of the chest, and the dilatation and contraction of the alæ nasi—symptoms that are never absent when the breath is oppressed. In some instances, towards the close of the disease, the head is violently retracted, while the lower jaw is depressed at each inspiration, and the patient lies, for a considerable time, with his mouth open, and gasping for breath, before death closes the scene.

"The respiration, though generally wheezing, is not always so. In some cases there is no wheezing at any period of the disease, and in others, though the breathing is loud and audible, the sound seems to proceed rather from some impediment offered to the air in its passage through the nostrils, than from mucous accumulation in the bronchia. It is difficult by description to convey an accurate idea of the sound to which I allude. I would call it a *dry* sound, in contradistinction to the other, which may be termed *humid*. It seems to be owing to a straitening of the air-passages, occasioned by the inflammatory turgescence of the membrane by which they are lined. When secretion takes place, this turgescence is in a great degree removed, and then the sound, from being dry, becomes moist and wheezing.

"In some instances the sound emitted in respiration seems to be a compound of the two sounds already mentioned, and to proceed, partly from nasal, and partly from bronchial obstruction. In the great majority of instances, however, the sound is distinctly wheezing, varying in degree from that of simple crepitus up to the loud and mucous rattle."*

The cough, which is generally short and dry, is most frequent at the beginning, and becomes less so as the disease advances. Towards the close, it is occasionally suspended altogether. A return of cough after such suspension

---

* "The sound here described, though in most cases abundantly evident to the naked ear, is heard with much more distinctness when we have recourse to the employment of the stethoscope. By means of this instrument we are also enabled to ascertain whether the inflammation is confined to the mucous membrane; for when the substance of the lung is affected, this is sufficiently indicated by the indistinctness or absence of the respiratory murmur in that portion which is the seat of disease. If in the exposition which I have given of the symptoms of this disease I have not mentioned the stethoscope, my omitting to do so has not arisen from any want of confidence in the indications afforded by this instrument, the value of which I can duly appreciate, but solely because I have not yet attained to that precision in its use which would justify me in speaking of it as a mean of diagnosis."

is to be considered a good sign. Young children swallow the expectoration, of course. The fever attending this disease runs extremely high.

"Intense heat of skin, dryness of the lips and nostrils, loaded white tongue, excessive thirst, and a pulse from 168 to 200, have been present in many instances; and to these symptoms, as I have already mentioned, have been superadded restlessness and jactitation, with moaning and starting out of sleep. In some cases the child can only be tranquillized by being carried about in his nurse's arms. As soon as he is laid on her knee, or in his cradle, the restlessness and crying will return, and continue until he is again put in motion."

The pulse, at the beginning, is generally hard, and sometimes full—during its progress it becomes more feeble—and towards the close, is usually imperceptible. Though the stomach is occasionally irritable at the commencement, it is often very difficult to produce vomiting towards the termination. This insensibility to the action of emetics has also been observed in croup. The same insensibility extends to the skin, and blisters often fail in exciting inflammation. The bowels are sometimes confined—at others, relaxed, with thin, green, slimy stools.

"I have mentioned that the countenance, though for the most part pallid, becomes more or less of a livid hue as the disease advances. The lividity is most remarkable in the lips and cheeks, but sometimes the palpebræ display a purplish, arborescent appearance, and the veins of the forehead are turgid with blood."

So far the description applies to cases that go on to a fatal termination. Let us see what is the series in more favourable cases. The cough becomes less frequent, and more loose-sounding—the breathing less hurried and wheezing—the fever subsides—the countenance resumes its healthy expression—sleep is obtained—the fever continues so long as there is any considerable affection of the respiration.

"With regard to the duration of this disease, when an unfavourable event takes place, it is generally upon the eighth or tenth day. When the case terminates in recovery, the disease is seldom prolonged beyond a week. In most instances the convalescence can be dated from the fifth, sixth or seventh day. Sometimes after the acute symptoms have subsided, more or less of cough, with wheezing respiration, will remain, and continue perhaps for weeks, the disease thus passing into the state of chronic bronchitis."

When chronic bronchitis is of long standing the expectoration becomes puriform—emaciation and hectic set in—and the little patient dies, with many of the symptoms of phthisis. On opening the body, however, we find that the disease exists principally in the mucous membrane, the substance of the lungs being generally unaffected.

This disease is much more prevalent in Winter and Spring than in any other season. Its cause cannot always be traced; but it is usually owing to exposure to wet and cold—hence it is more prevalent among the poor than the rich.

"The morbid appearance most frequently met with is an increase in the solidity of the lung, varying in degree from that of the slightest sanguineous congestion up to complete hepatization. This increase of solidity or induration is not equally great in every part of the lung. The inferior and posterior portion of the lung is in general the part principally affected; and it frequently happens that, while the upper portion is in a healthy state, or merely a little more congested than natural, the inferior portion is completely hepatized. It would appear as if the morbid process, commencing in the lower part of the lung, had completed its course there before the superior portion had advanced beyond the stage of sanguineous congestion. By hepatization I mean that state of the lung which is characterized by a purplish red colour externally, a bright red colour and granular appearance when the lung is incised, and a total absence of crepitus, the lung feeling firm and solid, and sinking in water. This state corresponds with the second degree of inflammation of the lung, as described by Laennec. The first degree of inflammation, according to the same author, is the state of sanguineous congestion above-mentioned. Laennec's third degree of inflammation, or that which is characterised by a purulent infiltration of the lung, I have not had an opportunity of observing. When the first degree of inflammation, or that of sanguineous congestion, prevails, it is generally combined with more or less of serous effusion into the interlobular tissue of the lung; but where the lung is hepatized its section appears dry and granular, and very little serous, or any other kind of effusion, escapes."

Along with this hepatization of lung which is *always* found after death, there is almost always more or less of inflammation observed in the mucous membrane of the bronchia—in some cases of the trachea. The more intense the inflammation of the mucous membrane, and the more considerable the effusion into the bronchia, the less general is the induration of the lungs, and *vice versâ*. With regard to the abdominal viscera, besides enlargement of the mesenteric glands, and tubercular depositions in the peritoneum, the liver was sometimes found pale, in some instances congested, enlarged, and with adhesions to neighbouring parts. Dr. C. thinks it probable that the inflammation usually commences in the mucous membrane, and is propagated thence to the substance of the lungs.

The danger in this disease is always proportioned to the violence of the symptoms, and their duration before proper treatment has been employed.

"The more intense the fever, the more hurried, laborious, and wheezing the respiration, and the greater the inability to expectorate the mucus accumulated in the bronchia, the more unfavourable the prognosis, and *vice versâ*. When lividity of the countenance with coma, and a weak, rapid, and intermitting pulse set in, the event is almost uniformly fatal."

It is consolatory to think that, if proper treatment be early put in force, the event will seldom be unfortunate. The fatal termination appears to be owing either to the suffocation occasioned by the accumulation of fluids in the bronchia—or to that indurated state of lung termed by Laennec hepatization, which renders the organ impermeable by the air.

The therapeutical objects would appear to our author to be threefold—*first*, to arrest the inflammation, before effusion has taken place—*secondly*, where effusion has obtained, to prevent its increase—*thirdly*, to adopt such measures as may promote absorption or expectoration.

To accomplish the first object bleeding, of course, is our sheet-anchor, and when early employed will strangle, as it were, the inflammation.

"In this early stage the breathing is loud and distinctly audible; it is not wheezing, the former sound arising from the straitening of the air passages, occasioned by the turgid state of their mucous membrane. It rarely, however, happens that our assistance is required before a certain degree of effusion has taken place, as is evinced by the wheezing of the respiration. We can seldom, therefore, expect to conduct the disease to a favourable termination, except through the medium of a free expectoration, which is, indeed, the most usual way by which the inflammation of the bronchial membrane, or of the substance of the lungs, resolves itself."

Dr. C. observes that, too much importance is often attached to the period of dentition, in the diseases of children, and the stomach and bowels are often exclusively looked to, when the lancet ought to be freely used. "It is as great a mistake to suppose that children do not bear bleeding well, as that the diseases to which they are liable do not require it." Here our author takes a very proper opportunity of questioning a dogma laid down by the present Dr. Hamilton, of Edinburgh, and who informs us that "bleeding by leeches, which some recommend in this (catarrhal fever) and other febrile affections of infancy, is, according to my experience, most injurious."* We confess we should attach exceeding little importance to the opinion of any man who could enunciate such a sweeping dogma, so repugnant to the daily observation of every practitioner in Great Britain.

The earlier the blood is drawn, the less occasion will there be for repetition —but it must be repeated till the symptoms are relieved.

"In general from two to three ounces in an infant between six and twenty months will be sufficient. In a child about two years of age from three to four ounces may be abstracted; and when the age is above four, about five, six, or eight ounces may be drawn, according to circumstances. In an infant under six months, though general blood-letting may often be required, the application of three or four leeches to the back of the hand or foot will for the most part answer the purpose, where a vein, which is frequently the case, cannot be found. It is better to apply the leeches to the hand or foot than to the thorax, for when they are applied in the latter situation, it is difficult to stop the bleeding after they have fallen off; and instances have occurred in which a fatal hæmorrhage has been the consequence of a continual oozing from four or five leech-bites. When they are applied to the hands or feet, the bleeding can be easily stopped by placing compresses of lint over the bites, and securing them, as after the operation of V. S., by a bandage. As far as my observation goes, leeches applied to the extremeties are nearly as efficacious in removing local inflammation in infants, as when applied in the vicinity of the part affected. They seem to produce the effect of a general blood-letting, as the face and lips become pale, the pulse faulters, and syncope occasionally takes place, followed by vomiting. These effects are apt to be produced when general V. S. is carried to a considerable extent, and sometimes a state of nervous agitation and general commotion is induced, which, if not speedily removed, may terminate in death. The best remedies in a case of this kind are the horizontal position, cool air, and a drop or two of the tincture of opium."

The blood of children in this disease does not usually shew the buff or cup. If it is desirable to have blood from a vein, the jugular will always supply it—generally a vein in the back of the hand. There is a point in this inflammation beyond which we dare not go on with blood-letting. When an abundant ef-

* Hints for the Treatment, &c. p. 84.

fusion has taken place into the bronchia, and when, as generally happens, this state is combined with more or less of collapse, the abstraction of even a small quantity of blood may be attended with fatal prostration. Expectoration—free and copious expectoration, offers the only chance of success in such cases. Even local depletion is then hazardous, and blisters should be substituted. As auxiliaries to the lancet, Dr. C. places much reliance on purgatives and emetics. In the early stage, before much mucous effusion has taken place, purgation lessens the quantum of circulating fluids, by restoring the secretions from the bowels; but, as in the case of bleeding, there is a limit to their employment. Our author prefers calomel and jalap; and after the bowels are well cleared, he keeps up the action by small doses of calomel and ipecacuan (one grain of each) every second, third, or fourth hour. He has seen decided relief from an antimonial emetic, when the lungs were much oppressed.

"Though Dr. John Clarke is so apprehensive of the employment of antimonials in the diseases of children, as far as my observation goes, I think his fears are in a great degree unfounded. I have used tartar emetic in many of the acute affections of children, and even of infants; and though I have repeatedly observed its good effects, I do not recollect a case in which I had occasion to regret its employment."*

After these remedies, blisters are to be applied; but care should be taken not to let them remain on longer than three or four hours, otherwise much irritation is produced. In infants of irritable habits, it is proper to weaken the emplastrum lyttæ, by mixing with it an equal quantity of emplastrum ceræ. In the advanced stage, when debility is great, and suffocation threatening, we must exhibit stimulants, of which, the carbonate of ammonia is the best. The decoction of seneka forms a good vehicle. When the prostration is extreme, wine and other cordials must be given, though they offer but very faint hopes of success.

Cases are minutely detailed by our author; but having portrayed his principles very fully, we need not enter on an examination of the facts on which they are founded.

---

*"In the peripneumonia of adults I have known the best effects to follow the administration of tartar emetic in large doses, as recommended by Laennec and other continental physicians. As the remedy, however, when so used, not unfrequently proves harsh and violent in its operation, I have been unwilling to employ it in the peripneumonia of children, except in the dose and manner above mentioned."
